# Vaccines as treatment for melanoma: an update review

**DOI:** 10.3389/fimmu.2026.1724490

**Published:** 2026-06-09

**Authors:** Jinjue Zhang, Qing Cao, Ying Cen, Ling Zhang, Junjie Chen

**Affiliations:** 1 Department of Burn and Plastic Surgery, West China Hospital, Sichuan University, Chengdu, China; 2 Department of Burn Plastic and Wound Repair, Jiujiang City Key Laboratory of Cell Therapy, Jiujiang NO.1 People’s Hospital, Jiangxi, China; 3 College of Polymer Science and Engineering, Sichuan University, Chengdu, China

**Keywords:** clinical trials, melanoma, nucleic acid vaccines, peptide vaccines, tumor vaccines

## Abstract

**Background:**

In past three decades, the development of therapeutic vaccines has been a major focus in melanoma research. This strategy seeks to prime the immune system to combat the cancer, ideally leading to the eradication of recurrent disease or a delay in its progression. To date, clinical trials have assessed multiple vaccine methodologies, but the evidence for a clear clinical impact remains ambiguous.

**Data sources:**

We retrieved relevant clinical trials published as of 1990 through electronic databases, including PubMed, the Cochrane library and ClinicalTrials.gov. The key search terms were “melanoma,” “vaccine,” and “clinical trials”.

**Study selection:**

Publications that met the following criteria were included as follows: (i) melanoma patients included; (ii) clinical trial registered; (iii) described the patients’ outcomes; (iv) were full-text articles published in English.

**Results:**

A total of sixteen distinct methodologies have been evaluated in randomized phase III clinical trials. Among these, Talimogene laherparevec (T-VEC) is the sole vaccine that has received approval from the U.S. Food and Drug Administration (FDA) for melanoma treatment. Most vaccine strategies demonstrated acceptable safety profiles and elicited immune responses, but they exhibited limited clinical efficacy. When integrated with other immunomodulatory agents, their therapeutic potential appears significantly enhanced.

**Conclusions:**

Within future therapeutic frameworks, melanoma vaccines are envisioned to play a key role by inducing and amplifying a targeted T-cell response against cancer. This approach is particularly suited for combination with other immunotherapies that neutralize the resistance mechanisms found in the tumor microenvironment.

## 1 Background

Melanoma is recognized as the most aggressive and deadly form of skin cancer, characterized by its rapid progression and high metastatic potential (1). Surgery continues to serve as the primary intervention for localized, early-stage disease (2). But for patients with advanced stage, chemotherapy and radiotherapy offered limited response rate of merely 10%, low overall survival (OS) and strong adverse side effects (3). Patients suffered frequent recurrence and metastasis. Immunotherapy has shown great efficacy in advanced melanoma treatment. Adjuvant therapies approved by the FDA for advanced melanoma include blockade of programmed cell death protein 1 (PD-1) and blockade of cytotoxic T-lymphocyte-associated protein 4 (CTLA-4). The objective response rate (ORR) has increased to 61% with the treatment of these immune checkpoint inhibitors (ICI). Despite this progress, a substantial proportion of patients do not respond to these therapies, highlighting a persistent demand for more effective systemic adjuvant options.

**Figure 1:**
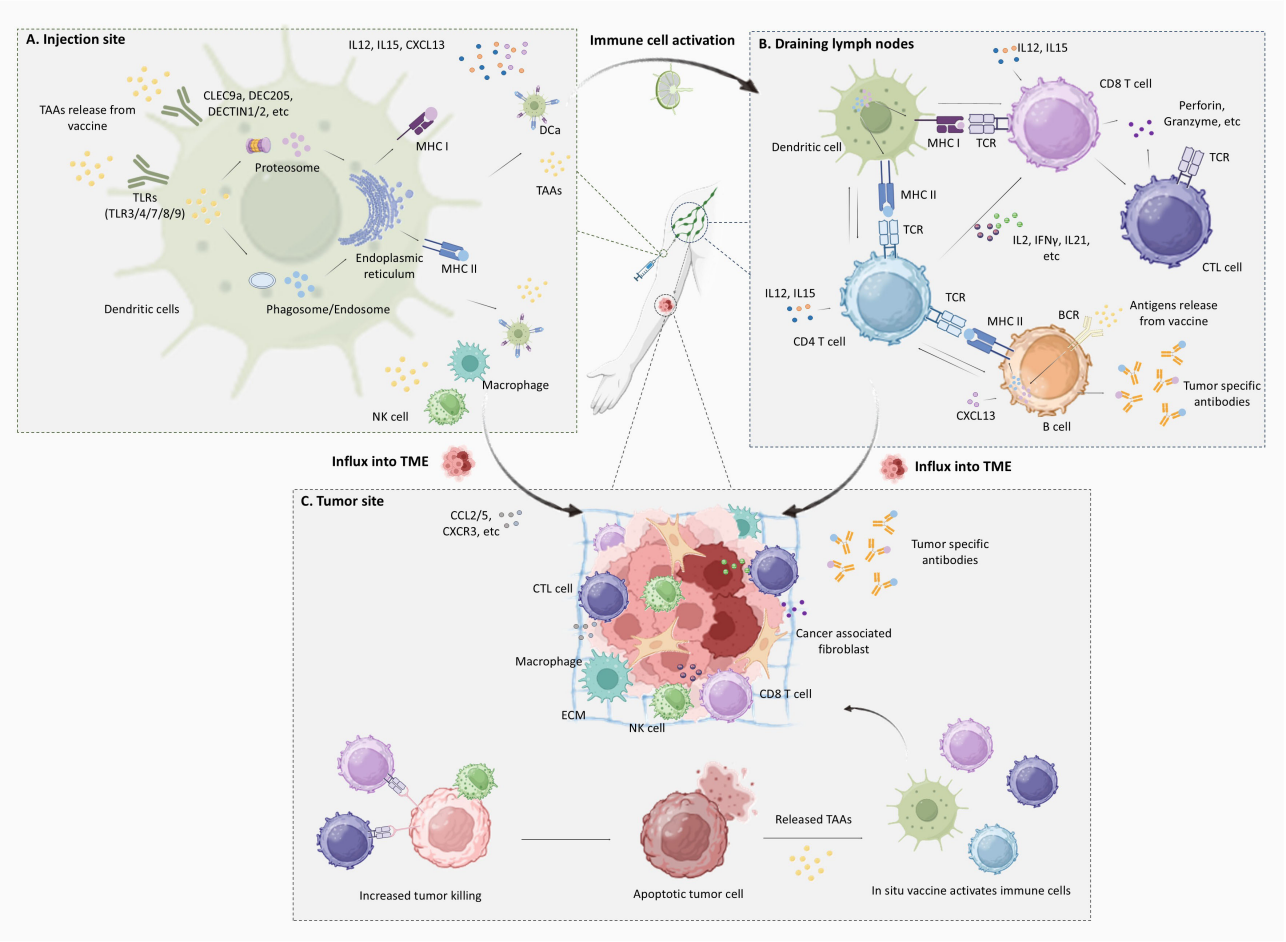
Anti-tumor process of tumor vaccine(5). **(A)** Tumor associated antigens released from vaccine activate antigen presenting cells (APCs), like dendritic cell (DCs). The diverse DC lineage expresses a broad array of pattern recognition receptors (PRR). PRRs include Toll like receptors (TLRs), which are present as transmembrane proteins to directly detect tumor associated antigens (TAAs) externally. CLEC9A/DNRG1 and DEC205/CLEC13B can facilitate phagocytosis of dead and dying cells. For MHCI, the proteasome process endogenous proteins into short peptides, transports into the endoplasmic reticulum (ER). For MHCII, proteins are processed into smaller peptides in phagosomes and endosome. In the ER, peptides are further processed and editors before transport to the cell surface. The activated DCs mature, upregulating phagocytosis, antigen presentation and co-stimulatory molecules, and migrate to a draining lymph node (dLN); lymph drainage also transports local antigens to the dLN. **(B)** In dLN, the activated DCs present MHCII restricted peptides to CD4^+^ T cells and cross-present antigen on MHCI to activate CD8^+^ T cells. Cytokines from DCs can shape CD4^+^ Helper T cell differentiation. CD4^+^ T cells potentiate CD8^+^ differentiation via IL2, IL21, and IFNγ. Activated CD8^+^ T cells produce cytokines and differentiate into cytotoxic T lymphocytes (CTLs). CD4^+^ T cells that recognize B cells can provide co-stimulation to support affinity maturation, antibody class switching and plasma cell differentiation. **(C)** Then vaccine specific immune cells migrate to the tumor, where it recognizes and destroys a cancer cell, releasing DAMPs that stimulate new DC maturation and phagocytoses tumor cell remnants, including non-vaccine targeted neoantigens. Tumor associated antigens (TAAs), Toll-like receptors (TLRs), Natural killer (NK), Activated dendritic cell (DCa), Cytotoxic T lymphocyte (CTL), peptide bound multi-histocompatibility complex (p:MHC), T Cell Receptor (TCR), B Cell Receptor (BCR), Extracellular matrix (ECM).

Supported by numerous anecdotal accounts of spontaneous tumor regression, melanoma is known for its inherent immunogenicity and thus has been the focus of immunotherapeutic vaccines (4). Antitumor vaccines are engineered to achieve several goals: triggering specific immune recognition, prompting tumor regression, eliminating residual malignant cells, establishing durable immunological memory, and minimizing off-target or toxic effects. Following administration, the vaccine comprising tumor-associated antigens (TAAs) is taken up by resident antigen-presenting cells (APCs), most notably dendritic cells (DCs), at the injection site. This uptake is facilitated by the adjuvant-induced inflammatory milieu, which promotes APC maturation and endows them with the capacity to process the exogenous antigens into immunogenic peptides. These mature APCs then migrate via afferent lymphatic vessels to the draining lymph node, the critical nexus for T cell priming. Within the lymph node, APCs present the processed antigenic peptides on major histocompatibility complex (MHC) molecules to naïve T cells, and drives the clonal expansion and differentiation of antigen-specific CD8^+^ cytotoxic T lymphocytes (CTLs) and CD4^+^ helper T cells. Following egress from the lymph node, these CTLs traffic through the peripheral blood, surveilling tissues until they infiltrate the tumor site. Upon encountering their cognate antigen presented on MHC class I molecules by tumor cells, CTLs execute their effector function by releasing cytotoxic granules containing perforin and granzyme B, thereby inducing target cell apoptosis (5) (**Figure 1**).

By augmenting immune responses directed against melanoma, such vaccines represent a promising strategy to prevent cancer recurrence. To date, Talimogene laherparepvec (T-VEC), an oncolytic viral therapy, stands as the sole vaccine approved by the FDA for advanced melanoma. Cancer vaccines come in different forms, including tumor-specific vaccines rely on peptides, proteins, nucleic acids, or carbohydrates, and non-tumor specific vaccines using inactivated whole tumor cells or antigen-presenting cells. Various methods have been used to enhance vaccine potency, including the identification of optimal target antigens and the incorporation of highly immunogenic adjuvants. This review concentrates on the principal vaccine strategies being investigated for melanoma and examines those that have progressed to clinical trial evaluation.

## 2 Antigen-undefined vaccines

A broad category of cancer immunotherapies comprises non-antigen specific vaccines, which are typically whole-cell based and do not target a single, defined antigen.

### 2.1 Tumor cell vaccines

In early studies, tumor oncolysates or irradiated whole tumor cells were used as vaccines and were well-studied in many phase III clinical trials (6, 7). Melacine was the first studied tumor oncolysate vaccine in patients with metastatic malignant melanoma in 1990s’ (8). Durable disease stabilization was observed in 10 to 20 percent of participants in all trials. However, a phase III multicenter study found that adding low-dose cyclophosphamide to Melacine produced equivalent outcomes in both response and survival to a four-drug chemotherapy protocol (9, 10). A correlation between the expression of human leukocyte antigen (HLA) class I and clinical outcomes was identified (11). Individuals who express the HLA-A2 and/or HLA-C3 alleles were associated with more favorable clinical results (12). Due to modest induction of tumor-associated antibodies induced by simple use of tumor cell vaccine (6, 7), further researches were prompted into strategies for enhancing their immunogenicity. One such innovation was M-Vax, an autologous vaccine created by conjugating a patient’s own tumor cells with the hapten dinitrophenyl. In clinical trials for stage III-IV melanoma, this preparation was administered intradermally alongside the Bacillus Calmette-Guérin (BCG) adjuvant (13). There were 11/83 antitumor responses in stage IV and a five-year overall survival rate of 44% in 214 stage III patients, which appears favorable when compared to the approximate 20-25% survival rate with surgery alone (14). M-Vax got the launched approval in Australia but was withdrawn from the market due to the manufacturer’s financial limitations. Similarly, the allogeneic whole-cell vaccine Canvaxin™ also combined with BCG, was evaluated in patients who had undergone complete resection of stage IV melanoma. This investigation failed to demonstrate any significant improvement in outcomes compared to the BCG/placebo control, leading to the discontinuation of its clinical development (15).

Subsequent investigation in a phase I trial evaluated VACCIMEL (CSF-470), an allogeneic vaccine comprising four lethally irradiated (70 Gy) melanoma cell lines. This vaccine, when combining with a granulocyte-macrophage colony-stimulating factor (GM-CSF) adjuvant, was shown to elicit a strengthened cellular immune response (16). The same investigators initiated a phase II/III study, adjuvant GM-CSF and BCG were injected simultaneously with the CSF-470 in patients diagnosed with stage IIB–III melanoma (17). In this study, after 5 years, a longer distant metastasis-free survival (DMFS) was observed in patients who received vaccine plus adjuvant GM-CSF and BCG (n=20, 96 months) than those treated with IFNa2b (n=10, 13 months) (18–20). In addition, patients with longer DMFS had a pronounced infiltration of CD8^+^ and CD20^+^ lymphocytes within the tumor microenvironment (p=0.004 and p=0.027, respectively), alongside the presence of peritumoral CD20^+^ aggregates devoid of CD21^+^ follicular dendritic cells (p=0.023) (20). A phase III trial was established but no relevant data found yet. Another vaccine named Seviprotimut-L, was developed from soluble antigens shed by three melanoma cell lines. This design intentionally excludes much of the extraneous cytoplasmic and nuclear material present in whole cells. A phase III trial enrolled 347 resected stage IIB-III cutaneous melanoma patients to assess its efficacy (21). However, relapse-free survival (RFS) was not significantly longer for seviprotimut-L in the full study population (22). The reason for differences in the clinical outcome between CSF-470 and Seviprotimut-L might be the usage of immune stimulating adjuvant, irradiation procedure or cell lines chosen in producing vaccines.

The new approach for enhancing the immunogenicity of tumor cells involves their genetic modification to express various cytokines (23, 24). The broadly known gene modified vaccine is GVAX for prostate cancer. Tumor cells were transfected to express GM-CSF. At least two phase III trials were initiated to evaluate its efficacy. But both trials had been terminated for mild clinical benefit comparing with chemotherapy. As for melanoma, several phase I/II studies have also investigated this methodology. Irradiated tumor cells were transfected with GM-CSF alone (GM-CSF TCV) (23) or TGF-β2/GM-CSF (TAG vaccine) (24). One stage IVa melanoma patient achieved a complete response (CR) after 11 TAG inoculations and remained disease free for 2 years (24). In stage III and stage IV patients receiving GM-CSF TCV, the median OS were 71.1 (95% CI, 43.7 to not reach (NR)) months and 14.9 (95%CI, 12.1 to 39.7) months, and the median progression-free survival (PFS) were 50.7 (95%CI, 36.3 to NR) months and 4.1 (95% CI, 3.0-6.3) months, respectively. Although autologous GM-CSF TCV augments antitumor immunity, and showed disease control in patients with stage III and IV melanoma, the overall disease control rate was 39.3% (95%CI, 27.1 to 52.7%). More clinical trials were needed to further investigate the benefit of GM-CSF expression autologous tumor cell vaccine compare with other treatments.

### 2.2 Dendritic cell vaccines

A distinct category of cellular vaccine utilizes autologous dendritic cells (DCs) that are primed ex vivo through exposure to specific cytokines or allogenic melanoma cells. Specifically, dendritic cell vaccines (DCVs) were produced by obtaining monocyte populations from autologous peripheral blood and cultured ex vivo with GM-CSF and IL4 to gain immature DCs, then incubated with irradiated autologous melanoma cells and finally administered via subcutaneous injection as a therapeutic vaccine (25). When evaluated in phase I/II clinical trials for advanced melanoma, this vaccine demonstrated more than two fold increase in the median 5-year OS and a 70% reduction of death risk comparing with single irradiated autologous tumor cell vaccine (25–28). Underlying this clinical performance, scientific investigations have revealed that the metabolic state of DCs is closely linked to the immunostimulatory capacity of DCV from individuals with cancer (29). To gain more specific anti-tumor immune activity, tumor antigen loaded-DCVs were developed and further studied, thus, most of non-specific DCVs were investigated in only phase I/II trials (30, 31). Other types of immune cell-based vaccines were merely studied.

## 3 Antigen-specific vaccines

A primary limitation of non-antigen-specific vaccines was their lack of a precisely characterized immunological objective. Furthermore, this strategy involved the concurrent delivery of a vast array of antigens, which could be potentially detrimental, immunologically insignificant, or could dilute the immunodominance of beneficial, protective epitopes. This understanding has driven the progression toward antigen-specific vaccine platforms, with the predominant aim being the creation of multi-valent formulations that concurrently engage several well-characterized immunogens.

### 3.1 Cancer-associated membrane carbohydrates

Cell-surface glycans, such as MUC1, globo H, and the α-gal epitope, have emerged as candidate targets for immunotherapeutic approaches in several cancer types. The design of α-gal glycolipids was conceived to facilitate the *in-situ* presentation of α-gal epitopes on malignant cells (32). This process effectively transforms the tumor into an endogenous vaccine, which is then opsonized by naturally occurring anti-Gal antibodies for uptake by antigen-presenting cells (APCs), bypassing the necessity for ex vivo modifications (32). A phase I clinical trial enrolled eligible patients with unresectable metastatic melanoma and a baseline serum anti-Gal titer of ≥1:50, administering two intratumoral injections of α-gal glycolipid at a four-week interval. Analysis revealed that three of eight evaluable patients exhibited upregulated PD-L1 expression on infiltrating macrophage/DC-like cells, accompanied by a markedly increased frequency of tumor associated antigen (TAA) pentamer^+^ T cells. However, immune cell infiltrations at injection site between pre- and post-treatment merely changed. The reason why treatment efficacy was low in some patients remains unknown but might related to treatment dose, or usage of combination treatment with immune checkpoint blockade.

The GM2 ganglioside is a tumor-associated antigen highly prevalent in melanoma. An investigational vaccine combining this antigen with the KLH conjugate and QS-21 adjuvant (GM2-KLH/QS-21), induced IgM and IgG antibody responses (33). A phase III study was conducted in 1314 patients with surgically resected stage II cutaneous melanoma, which evaluated the clinical benefit of GM2-KLH/QS-21 versus observation alone. However, analysis at the four-year mark, the vaccination cohort experienced a decreased RFS rate and OS rate (34). Consequently, GM2-KLH/QS-21 vaccination demonstrated no therapeutic benefit for patients with stage II melanoma and was not further studied.

### 3.2 Protein vaccines

Research on recombinant protein vaccines has established their utility to treat infectious diseases (35). Based on this approach, a vaccine comprising a chaperone complex of Hsp110 and gp100 was developed, demonstrating both antitumor effects and extended survival in mouse models of melanoma (36). This preclinical result led to a small phase I trial in which the human counterpart, a recombinant Hsp110-gp100 vaccine, was administered intradermally to individuals with unresectable advanced melanoma (37). The study employed a dose escalation design with 3 inoculations over 43-day period to evaluate toxicity, immune activation, and tumor response (37). Findings indicated modest clinical activity at that the lower doses of 30 and 60 mcg, with one patient achieving a partial response, one exhibiting stable disease, and six experiencing progression (37). Immunological analysis confirmed the activation of peripheral CD8^+^ T-cells, suggesting that combining this vaccine with established immunotherapies might potentially enhance its clinical efficacy. The phase I/II trials investigated a vaccine based on the New York Esophageal squamous cell carcinoma-1 (NY- ESO-1) protein, delivered either alone or in conjunction with various immune adjuvants such as poly-ICLC, incomplete Freund’s adjuvant (IFA), Ipilimumab or ISCOMATRIX (38–40). These regimens successfully elicited robust immunity, including NY-ESO-1-specific CD4^+^ and CD8^+^ T-cell responses (38–40). Despite effectively generating antigen-specific immunity, the vaccination did not translate into a statistically significant improvement in survival outcomes including OS and RFS (38–40). Consequently, NY-ESO-1 vaccine therapy has not advanced to more extensive clinical testing.

A phase 1/2 trial evaluated the immune-modulatory vaccine IO102-IO103, which was engineered to direct responses against immunosuppressive cells and tumor cells expressing indoleamine 2,3-dioxygenase (IDO) and PD-L1. This agent was delivered via subcutaneously injection concurrently with nivolumab (41). Long-term follow-up data revealed an ORR of 80%, with half of the 30 patients achieving CR. The median PFS was 25.5 months (95% CI 8.8 to 39), while median OS was not reached (95% CI 36.4 to NR) (42). Notably, robust response and survival rates were also observed in subjects with traditionally unfavorable prognostic factors, such as PD-L1 negative tumors, elevated lactate dehydrogenase (LDH), or M1c disease. Analyses of responders identified an influx of peripherally expanded T-cell populations into tumor sites, suggesting a mechanistic basis for the clinical activity. Based on these promising results, a phase III trial (NCT05155254) is now underway to compare the efficacy of IO102-IO103 combined with pembrolizumab against pembrolizumab monotherapy in advanced melanoma.

While protein-based formulations remain an active area of investigation for melanoma vaccines, it is not yet established whether they offer distinct clinical benefits compared to more simplified platforms, such as peptide-based or genetic vaccines.

### 3.3 Peptide vaccines

A range of class I and II MHC-restricted peptide epitopes has been originating from melanocytic differentiation antigens and cancer-testis antigens, including gp100 (43, 44), NY-ESO-1 (45, 46), MART-1 (47, 48), MAGE-A1 (49, 50), MAGE-A3 (50, 51) and MAGE-A10 (52, 53). Peptide vaccines have been administered to patients who have undergone complete resection of high-risk stage III-IV melanoma, with or without different immunostimulatory agents, such as (IFA, toll-like receptor (TLR) agonist, cytokines or anti-PD1 antibodies (54, 55). In early studies, single peptide vaccines were studied, such as gp100 using as an adjuvant to ipilimumab in a phase III trial (56). The outcomes indicated that the combination of ipilimumab with gp100 did not enhance therapeutic benefit; two- and three-year survival rates were 25% (24 of 95) and 25% (13 of 53) for ipilimumab monotherapy, compared to 19% (54 of 284) and 15% (24 of 156) for the combination arm (56). Conversely, a phase III study demonstrated that incorporating the same gp100 vaccine alongside IL-2 significantly improved clinical response rates and extended OS relative to IL-2 treatment alone (57). This marked discrepancy in outcomes likely reflects the divergent mechanisms of action of distinct immunotherapeutic classes, a hypothesis that warrants validation in future prospective clinical studies.

Later, the polypeptide vaccines [12MP (54, 55, 58), PV (59), 6MPH (60, 61), LPV7 (62)] have been found to be both safe and immunogenic in several early phase studies. However, a subsequent large phase III investigation involving 815 patients with resected stage IV or high-risk stage III melanoma, stratified by HLA-A2 status, failed to demonstrate a significant clinical benefit (63). Participants were randomized to receive either the peptide vaccine, GM-CSF, both agents, or a placebo. The final analysis revealed no statistically significant improvement in either OS (p=0.60) or RFS (p=0.71) for the active treatment arms (63). In this trial, patients with positive (HLA)-A2 status receiving vaccination of 13 cycles unless disease progression or unacceptable toxicity occurred. Those who relapsed before completing the initial series were offered an additional six cycles (63). In a subgroup analysis, a different trend was observed. No survival difference was observed for stage IIIA or IIIB patients. In contrast, subjects with stage IIIC/M1a disease who received the PV vaccine showed a longer median RFS (15.2 vs. 9.7 months; P = .040) and a strong trend toward improved OS (91.1 vs. 39.1 months; P = .128). Conversely, for patients with M1b/M1c disease, the vaccine was associated with inferior outcomes, including shorter RFS (5.5 vs. 20.9 months; P = .018) and OS (32.8 vs. 72.4 months; P = .288) (63). Several factors may account for these disparate results, including variability in patients’ baseline immune competence, the potential immunological irrelevance of the selected vaccine targets, the choice of adjuvant, and tissues related to vaccine delivery. Indeed, the presence of pre-existing immunity does not guarantee its therapeutic potency. The T-cell populations expanded by these specific peptides may have lacked genuine antitumor efficacy.

Recent years, a vaccine named UV1, composed of three synthetically generated long peptides derived from human telomerase reverse transcriptase (hTERT) was studied (64, 65). This design is predicated on the prediction that these peptides harbor numerous epitopes recognizable by various HLA alleles, positioning UV1 as a potential universal, off-the-shelf therapeutic vaccine that does not require pre-selection of patients based on their HLA type (64, 66). the induction of an immune response against these peptides has been correlated with promising clinical outcomes (ORR: 57%, CR:33%, 4-year OS: 69.5%, median PFS: 18.9 months in advanced melanoma patients) (66, 67). Immunological analyses confirmed these clinical benefits were accompanied by robust Th1 polarization, evidenced by the secretion of interferon-γ, tumor necrosis factor-α, and IL-2 (66, 67). A randomized phase II trial investigating UV1 in conjunction with ipilimumab and nivolumab is currently in progress.

A subsequent development involves personalized peptide vaccines, which are tailored from the unique neoantigens (nAgs) of an individual patient’s tumor (68). These nAgs represent mutated self-antigens originating from somatic mutations exclusive to malignant cells, rendering them absent in healthy tissues. Consequently, the immune system identifies these neoantigens as foreign, potentially initiating a potent and highly selective antitumor response. Several phase I trials provided evidence that such vaccines can induce T-cell immunity directed against the targeted neoantigens (45, 64, 65, 67, 69). However, these early investigations demonstrated limited therapeutic efficacy, with minimal impact on objective response rates or delaying disease progression (45, 64, 65, 67, 69). The suboptimal clinical performance may be attributed to the heterogeneous peptide mixtures employed, not all of which are functionally capable of expanding cytotoxic T-cell populations in every recipient. This lack of consistent, robust benefit largely confined further development of such vaccines beyond initial trial. However, a phase II trial investigated an AI-generated personalized neoepitope vaccine EVX-01, in combination with pembrolizumab for unresectable or metastatic melanoma (70). This approach successfully stimulated durable, vaccine-specific T-cell responses in all treated patients (71). The one-year follow-up data reported objective clinical responses in 11 out of 16 patients, resulting in a 69% ORR (72). These promising outcomes help substantiate the accuracy and predictive capability of the proprietary AI-based platform used for vaccine target identification.

### 3.4 Antigen-loaded DC vaccines

The unique capacity of DCs to internalize external antigens and initiate T-cell responses has prompted numerous clinical studies. These investigations involve loading DCs with various immunogens, such as nucleic acids, peptides, or whole proteins, for therapeutic delivery. One type of DC vaccine was produced by electroporating or pulsing the autologous DCs with tumor specific mRNA or HLA-restricted antigens *in vitro*. Although a large amount of phase I/II trials studied the immunogenicity, safety, and clinical impact of such DC-based vaccines, most have demonstrated only marginal enhancements in T-cell reactivity or patient outcomes (73–78). In a single arm phase II study, patients with stage IV or recurrent stage III melanoma received subcutaneous inoculations of autologous dendritic cells that had been pulsed with lysates from irradiated autologous tumor cells (known as DC-TC, Melapuldencel-T, or eltrapuldencel-T), suspended in a GM-CSF solution (79). The results indicated a two-year survival of 72% for the vaccinated cohort versus 31% in control, and a 5-year survival of 50% in vaccine group (79, 80). This vaccine was advanced to a phase III trial (NCT01875653) but was terminated for unknown reasons. In another phase III clinical trial, the DC vaccine, consisting autologous DCs loaded with tumor peptides and overlapping peptide pools, was 2:1 (vaccine:placebo) randomly given to 148 patients with resected stage IIIB/C melanoma. Immunological monitoring confirmed that functional antigen-specific T-cell responses were detectable in 67.1% of assessed patients within the treatment arm, compared to just 3.8% in the control group (p<0.001) (81). However, while the adjuvant DC vaccination elicited detectable immunity and was well-tolerated, it did not translate into prolonged RFS for the participants (81).

Another type of DC vaccine involves loading a patients’ own DCs with lysates derived from their autologous tumor cells. This strategy aims to present a broad array of tumor-associated antigens, thereby inducing a polyclonal immune response capable of eliminating malignant cells while maintaining a favorable safety profile. A phase II investigation involving 33 metastatic melanoma patients reported clinical outcomes including one CR, two PR and six SD, resulting in a prospectively defined disease control rate of 27% (82). A separate phase I/IIa study of 25 metastatic melanoma patients added IL2 to DC vaccination treatment. The results indicated a significantly longer median OS for individuals receiving a higher frequency and number of doses (23.8 vs. 8.7 months, p= 0.004) (83). Further development led to the tumor lysate particle-loaded dendritic cell (TLPLDC) vaccine, which demonstrated improved disease-free survival (DFS) in the resected stage IV melanoma patients either (84–86). Thus, the same group compared TLPLDC with autologous tumor lysate particle only (TLPO) vaccine in another phase IIB trial (87). This study randomized 187 patients to receive either a placebo, TLPO or TLPLDC with or without GM-CSF. The analysis revealed that both the TLPLDC (without GM-CSF) and the TLPO vaccines were associated with improved DFS and OS (87). Owing to its more straightforward production process, the efficacy of the TLPO vaccine is slated for validation in a forthcoming phase III clinical trial.

### 3.5 Viral and bacterial vaccines

The inherent capacity of the immune system to identify and combat pathogenic microorganisms has inspired the use of viral and bacterial vectors to provoke antitumor adaptive immunity (88). A prominent example is T-VEC, an oncolytic vaccine based on a modified herpes simplex virus type 1. It is engineered to selectively replicate inside cancer cells and secrete GM-CSF. The expression of GM-CSF provides the critical ‘adjuvant’ signal needed for effective antigen presentation, turning the dying tumor cells into a personalized antigen source, thereby stimulating a systemic immune response against the tumor and function as an *in situ* cancer vaccine. Clinical studies demonstrated that T-VEC exhibits a favorable safety profile and achieved superior durable response rates (19.0% in the T-VEC arm and 1.4% in the GM-CSF arm, (unadjusted odds ratio, 16.6; 95% CI, 4.0–69.2; p < 0.0001)) and extended median OS (23.3 months (95% CI, 19.5–29.6) in the T-VEC arm and 18.9 months (95% CI, 16.0–23.7) in the GM-CSF arm) in treatment-naive individuals with stage IIIB to IV melanoma (89). The final OPTiM analysis confirmed these sustained efficacy benefits and ongoing tolerability compared to GM-CSF administration alone (90). It was then approved by FDA in the usage as a single agent in advanced melanoma (91). Recently, studies which combining T-VEC with radiotherapy, chemotherapy or ICI therapy were formed to analyze potential additional benefits.

The FDA approval and the great clinical benefit of T-VEC helped oncolytic viruses gain wide attention in cancer therapy. A powerful vaccine platform which boosts with non-human adenoviral vectors from Great Apes (GAd) and a Modified Vaccinia Ankara (MVA), has been shown to elicit potent and sustained T-cell immunity in the contexts of both infectious diseases and oncology (92). NOUS-PEV is a personalized cancer vaccine that utilizes GAd20 and MVA vectors to deliver 60 patient-specific neoantigens (93). Results from a phase I trial, which combined NOUS-PEV with pembrolizumab in treatment-naïve metastatic melanoma patients, revealed the induction of neoantigen-specific T cells in all evaluable participants. The observed best overall responses included 1 CR, 3 PR, 1 SD, and 1 PD. These findings indicate that the combined rigimen was safe, highly immunogenic, and exhibits promising anti-tumor activity.

IMM-101 is a therapeutic agent comprising heat-killed, whole cell *Mycobacterium vaccae*, which has been extensively evaluated in human trials across multiple indications. This immunomodulator has demonstrated a capacity to favorably alter immune activity while exhibiting a highly favorable safety profile, suggesting potential utility for individuals with cancer. A randomized, placebo-controlled phase I investigation assessed its safety and tolerability in patients diagnosed with stage III or stage IV melanoma (94). The study protocol involved a series of three intradermal injections administered over a 4-week period. The trial successfully established the 0.5-mg and subsequently the 1.0-mg dose levels as suitable for proceeding with further clinical development. Furthermore, during the post-treatment monitoring phase, clinical responses were observed in 15% of the participants with stage IV disease.

### 3.6 Nucleic acid vaccines

Nucleic acid vaccines represent a platform wherein plasmid DNA or messenger RNA (mRNA) sequences serve as delivery vehicles for genetically encoded immunogens. Their fundamental mechanism shares similarities with viral vector approaches. However, a key distinction lies in the absence of foreign viral gene expression. This characteristic confers several advantages, including an improved safety profile compared to bacterial or viral vectors, a minimal risk of insertional mutagenesis, and the avoidance of anti-vector immunity (95).

#### 3.6.1 Nucleic acid vaccines-plasmid DNA

DNA vaccines require intracellular processing, including transcription and translation, before dendritic cells can present these antigens to the immune system. The initial antigen selected for DNA vaccines development in melanoma was gp100. Two randomized phase I trials studied the safety, dose and immunologic response of gp100 DNA vaccine in stage IIB-IV melanoma patients and demonstrated enhanced antigen-specific T cell responses. Then, a huIgG1 antibody DNA vaccine (SCIB1), which targets both gp100 and TRP-2, has shown immunogenicity and anti-tumor benefit in combining PD-1 blockade in preclinical study (96). A subsequent phase I/II dose escalation study by intramuscularly injection to patients with metastatic melanoma at doses ranging from 0.4mg-8mg (97). The vaccine produced dose-dependent T-cell responses in 88% of participants, with no severe adverse events or dose-limiting toxicities reported (97). Among 15 patients with measurable disease, one achieved an objective tumor response, while seven exhibited stable disease (97). However, this trial was terminated for low product availability. Later, other types of antigens for DNA vaccines including tyrosinase, MART-1, and NY-ESO-1 were studied, but most of them were not efficient enough in phase I trials to support further exploration (98). Recently, a vaccine named IFx-Hu2.0, which expressed Emm55 protein fragment, was shown to activate both innate and adaptive immunity in animal models. A phase I clinical study administered this agent via intralesional injection in patients with unresectable stage III or IV melanoma (99). Notably, three out of four participants who were previously refractory to anti-PD1 therapy subsequently derived clinical benefit from a renewed course of anti-PD1 treatment. These findings suggest that this intralesional priming strategy is a feasible intervention, and the correlative immune data support its continued investigation to sensitize metastatic melanoma patients to an immune priming agent.

#### 3.6.2 Nucleic acid vaccines-mRNA

In contrast to DNA-based platforms, RNA vaccines bypass the need for nuclear transcription, enabling more direct expression of protein antigens and their subsequent processing for presentation on major histocompatibility complex (MHC) molecules. To date, two primary methodologies for direct mRNA injection have been documented. A liposomal RNA vaccine named FixVac (BNT111), which is designed to target four conserved, non-mutated tumor-associated antigens commonly expressed in melanoma, showed pivotal immunogenicity and antitumor effects in mouse model (100, 101). These findings supported its advancement into a phase I trial for individuals with advanced disease (102). Vaccine was intravenously administered either as a monotherapy or concurrently with anti-PD-1 antibodies. The vaccine proved effective in humans, stimulating potent CD4^+^ and CD8^+^ T-cell immunity against its target antigens. The combination treatment achieved better PR and SD than monotherapy (PR: 6/17 versus 3/25, SD: 2/17 versus 7/25).

Another mRNA vaccine, known as V940 (mRNA-4157), encoding 34 neoantigens specifically to tumor-mutanome and human leukocyte antigen type, was studied in a phase IIb study (103). The neoantigens for the vaccine are identified via a proprietary algorithm, the construct had previously shown a favorable safety profile and the capacity to elicit robust T-cell responses in preclinical models (104, 105). The mRNA-4157 vaccine was randomly given to 157 patients with completely resected melanoma as adjuvant to pembrolizumab therapy. Combination therapy of mRNA-4157 and pembrolizumab showed a manageable safety profile and prolonged RFS versus pembrolizumab monotherapy (18-month RFS, 79% (95% CI 69·0–85·6) versus 62% (46·9–74·3), respectively). These results provide evidence on using mRNA-based vaccine as adjuvant therapy in melanoma. Based on these promising results, a phase III registrational trial (NCT05933577) has been initiated.

## 4 Lessons learned to guide future antitumor vaccine approaches

The development of therapeutic vaccines targeting singular or multiple tumor antigens has constituted a major research focus in melanoma treatment for the past three decades (**Supplementary Table S1**). While most of these investigational agents have consistently demonstrated favorable safety profiles and signs of immune activation, they have progressed only to initial clinical testing. To date, sixteen distinct vaccine methodologies have advanced to randomized phase III clinical evaluation (**Table 1**). Among these, only T-VEC, gp100 peptide vaccine, and M-vax have successfully extended survival in patients with stage III-IV melanoma. In addition, mRNA-4157 plus pembrolizumab has recently exhibited a tolerable safety record and significantly extended RFS in high-risk stage II-IV melanoma patients, and is now recruiting patients in a phase III trial. Currently, T-VEC remains the sole therapeutic cancer vaccine approved by the FDA for advanced melanoma. The limited success of rest type of vaccines stems from an inability to generate sufficiently potent or durable T-cell responses necessary for sustained antitumor immunity, underscoring the critical need for innovative strategies to advance the field of cancer vaccine development.

**Table 1:** Phase III trials to assess antitumor vaccines in patient with melanoma.

Antigen specificity	Vaccine type	Vaccine	Antigen	Combination agents	Patients enrolled	Study title	Results and/or comment
Antigen-undefined	Tumor cell vaccines	Melacine	NA	IFN-A	47	Phase III trial of melacine plus interferon alfa-2b versus interferon alfa-2b in patients with disseminated malignant melanoma (NCT00002767)	No improvement in survival and objective response rate (9, 10).
		CancerVax™ vaccine (CANVAXIN)	NA	BCG	496	A Phase III Randomized Double-Blind Pivotal Trial of Immunotherapy With a Polyvalent Melanoma Vaccine, CancerVax™ Vaccine Plus BCG Versus Placebo Plus BCG as a Post-Surgical Treatment for Stage IV Melanoma (NCT00052156)	No improvement in RFS and OS (16).
		CSF-470 (VACCIMEL)	NA	BCG and rhGM-CSF	31	Randomized, Comparative Phase II/III Study Between Treatment With CSF470 Vaccine (Allogeneic, Irradiated) Plus BCG and MOLGRAMOSTIN (rhGM-CSF) as Adjuvants and Interferon-alfa 2b (IFN-ALPHA), in Stages IIB, IIC and III Post Surgery Cutaneous Melanoma Patients (NCT01729663)	Unknown status of phase III trial, significantly prolong DMFS with lower toxicity in phase II study (18).
		seviprotimut-L	NA	NA	347	A Multicenter, Double-blind, Placebo-controlled, Adaptive Phase 3 Trial of POL-103A Polyvalent Melanoma Vaccine in Post-resection Melanoma Patients With a High Risk of Recurrence (NCT01546571)	No improvement in RFS(22), but had efficacy in patients younger than 60 (22).
		M-vax	NA	IL2	297	Comparison of M-Vax Plus Low Dose Interleukin-2 Versus Placebo Vaccine Plus Low Dose Interleukin-2 in Patients With Stage IV Melanoma (NCT00477906)	Improvement in survival(13), and was approved in Australia in 2000, but withdrawn in 2002 due to financial constraints (106).
Antigen specific	Protein vaccines	GM2-KLH Vaccination	GM2	QS-21	1314	Adjuvant Ganglioside GM2-KLH/QS-21 Vaccination: Post-Operative Adjuvant Ganglioside GM2-KLH/QS-21 (BMS-248479) Vaccination Treatment After Resection of Primary Cutaneous Melanoma Thicker Than 1.5mm (AJCC/UICC Stage II, T3-T4N0M0), a 2-Arm Multicenter Randomized Phase III Trial vs. Observation (NCT00005052)	No improvement in RFS and OS (35).
		IO102-IO103	IDO, PD-L1	Pembrolizumab	407 (estimated)	An Open-label, Randomized, Phase 3 Clinical Trial of IO102-IO103 in Combination With Pembrolizumab Versus Pembrolizumab Alone in Patients With Previously Untreated, Unresectable, or Metastatic (Advanced) Melanoma (IO102-IO103-013 / MK3475-D18) (NCT05155254)	Active, not recruiting
	Peptide vaccines	MDX-1379 (gp100)	gp100	ipilimumab	676	A Randomized, Double-Blind, Multicenter Study Comparing MDX-010 Monotherapy, MDX-010 in Combination With a Melanoma Peptide Vaccine, and Melanoma Vaccine Monotherapy in HLA-A2*0201-Positive Patients With Previously Treated Unresectable Stage III or IV Melanoma (NCT00094653)	No improvement on ipilimumab efficacy and survival rates (57).
		gp100: 209-217 (210M) Peptide	gp100	IL2	185	A Phase III Multi-Institutional Randomized Study of Immunization With the gp100: 209-217 (210M) Peptide Followed by High Dose IL-2 vs. High Dose IL-2 Alone in Patients With Metastatic Melanoma (NCT00019682)	Improvement in PFS (58).
		PV	tyrosinase, gp100, and MART-1	GMCSF	815	A Randomized, Placebo-Controlled Phase III Trial of Yeast Derived GM-CSF Versus Peptide Vaccination Versus GM-CSF Plus Peptide Vaccination Versus Placebo in Patients With "No Evidence of Disease" After Complete Surgical Resection of "Locally Advanced" and/or Stage IV Melanoma (NCT01989572)	No improvement in RFS and OS (64).
		vaccine modified to express HLA A2/4-1BB	HLA A2/4-1BB restricted peptide epitopes	NA	50	Allogeneic Vaccine Modified to Express HLA A2/4-1BB Ligand for High Risk or Low Residual Disease Melanoma Patients (NCT01861938)	Unknown status (no efficient data found)
		Tumor peptide vaccines	MART-1, NA17-A, gp100, tyrosinase	NA	13	Randomized Phase III Study Of Adjuvant Immunization With The NA17.A2 And Melanoma Differentiation Peptites In HLA-A2 Patients With Primary Ocular Melanoma At High Risk Of Relapse (NCT00036816)	Terminated: low accrual
	Antigen-loaded dendritic cell or antigen-presenting cell vaccines	DC-TC	NA (tumor cell lysaates)	GM-CSF	4	Phase III, Randomized, Double-Blind, Multicenter Trial of Autologous Dendritic Cells and Irradiated Autologous Tumor Cells In Granulocyte-Macrophage Colony-Stimulating Factor (GM-CSF) vs. Autologous Peripheral Blood Mononuclear Cells (PBMCs) In GM-CSF for The Treatment Of Metastatic Melanoma (NCT01875653)	Terminated: unknown reasons.
		nDC vaccination	NA (synthetic tumor peptides)	NA	148	A Randomized, Double--Blind, Placebo-Controlled Phase III Study to Evaluate Active Immunization in Adjuvant Therapy of Patients With Stage IIIB and IIIC Melanoma With Natural Dendritic Cells Pulsed With Synthetic Peptides. (NCT02993315)	Generated specific immune responses and was well tolerated, but no improvement in RFS (82).
	Viral, bacterial and fungal vaccines	Talimogene laherparepvec (T-VEC)	NA	GM-CSF	436	A Randomized Phase 3 Clinical Trial to Evaluate the Efficacy and Safety of Treatment With OncoVEX^GM-CSF Compared to Subcutaneously Administered GM-CSF in Melanoma Patients With Unresectable Stage IIIb, IIIc and IV Disease (NCT00769704)	Provide durable CRs and prolonged survival(90), approved by FDA for melanoma treatment.
	Nucleic acid vaccines	V940 (mRNA-4157)	NA (neoantigens)	pembrolizumab	1089 (estimated)	A Phase 3, Randomized, Double-Blind, Placebo- and Active-Comparator-Controlled Clinical Study of Adjuvant V940 (mRNA-4157) Plus Pembrolizumab Versus Adjuvant Placebo Plus Pembrolizumab in Participants With High-Risk Stage II-IV Melanoma (INTerpath-001) (NCT05933577)	Recruiting (prolonged recurrence-free survival in previous study (104))

The shift toward personalized, precision oncology underscores the importance of integrating lessons from historical vaccine development. First, the choice of target antigens to form vaccines, the platforms of vaccines, and whether in combination with other immunotherapies might have direct impact on treatment efficiency. Tumor-associated antigens serve as essential signals that direct the immune system to recognize and eliminate cancer cells. Nonetheless, the clinical utility of vaccines can be compromised by tumor immune evasion mechanisms, such as antigen loss variants. Furthermore, the significant clonal heterogeneity within melanomas means that certain subpopulations may inherently lack the targeted epitope, thereby escaping immune recognition. While vaccines consisting of tumor lysates offer the advantage of presenting a broad antigenic repertoire, this approach carries the potential drawback of recruiting immunosuppressive cell populations or diluting the immune response against the most clinically relevant antigens. Thus, comparing to whole-tumor antigen vaccines and shared-antigen vaccines, the vaccines directing against tumor neoepitopes is more attractive. Vaccines which use more neoantigens, especially selected by bioinformatic methods showed better immune stimulating responses and prognostic outcomes. For instance, WTC vaccine CSF-470, protein vaccine IO102-IO103, personalized peptide vaccine EVX-01, and mRNA vaccine V940 (mRNA-4157). Besides, immune modulatory vaccines (IMVs) targeting immunosuppressive elements within the TME, such as IDO, PD-L1, arginase-1 (ARG1), and TGF-β represent an underexplored frontier (107). However, infrequently accessible of autologous tumor, the large amount of effort to develop individual-specific products, and the current limitations in the predictive accuracy of bioinformatic algorithms for epitope selection. Future research leveraging large-scale genomic datasets and advanced computational modeling may eventually enable the rational design of poly-neoepitope vaccines, overcoming these significant hurdles.

Advancements in antigen delivery systems have catalyzed significant progress in the design of modern vaccine platforms. We summarized the advantages and disadvantages of different vaccines in **Table 2**. The results across antigen-loaded DC vaccine trials have not been consistent. The challenges to commercialization and the complexities associated with manufacturing of DC vaccines may lead to less priority in its’ development. Peptide vaccines have the shortest processing route from injection site to APCs. In contrast, nucleic acid vaccines demonstrate superior efficacy in delivering antigens for MHC class I presentation. While DNA vaccines necessitate additional intracellular processing steps before epitope presentation by dendritic cells, their administration can be enhanced by techniques such as *in vivo* electroporation. Conversely, mRNA formulations can be systemically delivered using novel nanoparticle carriers, including lipoplexes, which efficiently target the vaccine to DCs residing in lymphoid tissues. A particularly compelling attribute of mRNA-based immunotherapies is their favorable safety profile. This platform holds considerable promise for stimulating coordinated innate and adaptive immunity, offering a framework that is adaptable for both personalized medicine and scalable manufacturing. Its inherent flexibility also permits extensive customization of antigenic targets, structural configurations, and combination strategies. Although this innovative field remains in a nascent stage of development, it holds substantial potential for creating novel and effective cancer treatment protocols.

**Table 2:** Advantages and disadvantages of different vaccines.

Vaccine platform	Advantages	Disadvantages
Tumor Cell Vaccines	Poly-antigenic: Presents a broad array of antigens, reducing chance of tumor immune escape.	Modest Efficacy: Limited clinical benefit (e.g., failed Canvaxin phase III trial). Safety Concerns: Potential risks from administering viable cells.
Dendritic Cell (DC) Vaccines	Potent Antigen Presentation: Uses professional APCs for robust immune activation.	Logistically Complex: Ex vivo manipulation is expensive, time-consuming, and requires specialized facilities. Difficult to Scale: Standardization is challenging. TME Limitations: Efficacy can be suppressed by Tregs and immunosuppressive tumor microenvironment.
Peptide Vaccines	Simplicity & Purity: Easy to synthesize and well-defined. High Specificity: Targets precise epitopes.	HLA Restriction: Efficacy limited to patients with specific HLA types. Low Immunogenicity: Often requires potent adjuvants to elicit a response. Mono-epitopic: Narrow focus can lead to immune escape.
Protein Vaccines	Multi-epitopic: Full-length proteins can be processed to present multiple epitopes.	Manufacturing Challenges: More complex to produce than peptides. Mixed Clinical Results: Promising early data often fails in late-phase trials (e.g., MAGE-A3).
mRNA Vaccines	Rapid & Scalable Production: Easily manufactured, especially critical for personalized approaches. Flexible: Can encode multiple antigens (shared or neoantigens). Strong Efficacy Data: Personalized neoantigen mRNA (mRNA-4157) + PD-1 inhibitor showed significant reduction in recurrence risk in phase IIb (leading to phase III trial).	Instability: Requires cold chain and complex delivery systems (e.g., lipid nanoparticles). Personalized = Expensive: Individualized neoantigen vaccines are costly and require advanced sequencing technology. Non-personalized = Generic: Off-the-shelf shared antigen mRNA (e.g., BNT111) is not unique to every patient's tumor.
DNA Vaccines	Highly Stable: More stable than mRNA, no cold chain required. Fast Production: Can be produced rapidly. Safe Profile: No risk of integration into host genome (theoretical risk low).	Poor Immunogenicity: Difficulty in generating strong systemic T-cell responses in humans. Delivery Challenges: Requires specialized devices (e.g., electroporation, gene gun) for effective cellular uptake. Limited Efficacy: Clinical trials (e.g., MART-1 DNA tattoo) have shown local but not systemic responses.
Viral Vector Vaccines	Highly Immunogenic: Viral backbone provides inherent "danger signals" (adjuvant effect). Efficient Delivery: Excellent at transducing cells and delivering genetic material.	Anti-Vector Immunity: Preexisting immunity or strong response to the virus can dominate, diverting immune focus away from the tumor antigen (cargo). Safety Concerns: Potential for insertional mutagenesis (rare). Complex Manufacturing: Difficult to produce at scale.

Based on previous studies, vaccines used as single agent showed rather modest clinical activity, indicating that vaccines alone are not sufficient to amplify and/or activate tumor-specific T cells in advanced melanoma. Effective immunization will most likely require integration with complementary immunostimulatory agents which can prime DCs and T cells, including IL2, GM-CSF, ICIs, BCG or PD1/PD-L1 therapy. Strategically designed combination regimens may even create an *in-situ* environment that prompts the patient’s immune system to generate its own endogenous “neoepitope vaccine” (108). However, a significant challenge lies in optimizing these multi-agent protocols, including identifying the most potent agent combinations and defining their optimal sequence, timing, and dosage. Further complexity arises from the functional specialization of DC subsets. The conventional type 1 DC (cDC1) population excels at cross-presenting antigens and activating CD8^+^ T cells, whereas the cDC2 subset is primarily responsible for initiating CD4^+^ T helper responses. Emerging evidence from studies combining chimeric antigen receptor (CAR) T cells with amplifying vaccines suggests a promising therapeutic blueprint. One potential strategy involves inducing initial remission with CAR T-cell therapy, followed by consolidation with ICIs to sustain long-term disease control. The advent of advanced analytical tools, including single-cell RNA sequencing and high-dimensional flow cytometry, will further accelerate this field by enabling the rapid characterization of vaccine-induced T-cell responses. This capability will help identify the most immunogenic vaccine candidates early, thereby preventing costly large-scale trials of less effective formulations.

Second, considering individual patient characteristics and tumor biology, including age, sex, and tumor stage may lead to more refined treatment strategies. Several vaccines showed better efficiency in female patients, younger patients, patients with positive HLA status and patients with locoregional disease. A growing body of evidence positions biological sex as a significant determinant of immunotherapy outcomes. Divergent immune landscapes between males and females, marked by variations in the functionality and prevalence of both antitumor and immunosuppressive cell populations, are likely influenced by a composite of hormonal and genetic factors. To develop more precisely tailored immunotherapies, future research must elucidate the concentration of sex hormones within the TME and define their impact on antigen presentation and T-cell priming within tumor-draining lymph nodes. Advancing age represents another crucial risk factor, with the natural decline in sex hormone production potentially linked to age-related immune-senescence. Despite these recognized variables, no clinical trials to date have been prospectively designed to evaluate sex-specific dosing or differential responses to immunotherapeutic agents. A comprehensive investigation into the interplay of hormonal and chromosomal factors is therefore essential to unravel the mechanisms driving persistent sex-based disparities in cancer immunity and treatment efficacy across the human lifespan.

Evidence showed that higher pre- and post- treatment concentrations of MMP-1, TRAIL, CXCL-11, and CXCL-13 were correlated with prolonged PFS (23, 109). Specifically, regarding T-VEC, the presence of TERT promoter mutations (particularly C228T and C250T variants) has emerged as a significant driver of clinical benefit and local control in patients receiving intralesional T-VEC (p=0.043 for response) (110). The adoption of personalized medicine, guided by predictive biomarkers, is poised to become a standard paradigm for enhancing clinical response and extending patient survival. One well-documented prognostic indicator in oncology is the presence of tumor-infiltrating lymphocytes (TILs). These cells are instrumental in executing antitumor immunity within the local microenvironment. Elevated TIL density is frequently linked to more favorable clinical outcomes and heightened sensitivity to immunotherapeutic agents across multiple cancer types. The immunosuppressive Tregs and MDSCs in TME can mediate T cell exhaustion, and may further dampen the antitumor immune response. Current strategies to overcome TME-mediated immunosuppression including combination with immune checkpoint inhibitors, or chemokine modulation, which aim to counteract immune evasion and enhance T-cell responses. A new strategy named immune modulatory vaccines (IMVs) which directly target TME antigens (TMAs) combined with anti–PD-1 therapy demonstrated that IMV can improve PFS in metastatic melanoma in a phase III study (107).

## 5 Conclusions

The primary objective of antitumor vaccination is to induce a targeted immune response capable of mediating tumor cell eradication. For more than three decades, a burgeoning research focus has been dedicated to creating vaccine-based interventions for melanoma. This endeavor is evidenced by over 192 clinical trials investigating a diverse array of methodologies, including whole-cell preparations, non-antigen-specific formulations, and antigen-defined platforms that employ proteins, peptides, viral or bacterial vectors, and nucleic acids to deliver immunogenic targets. To date, 16 of these strategies have progressed to randomized phase III testing. Among these, only three regimens, T-VEC combined with GM-CSF, gp100 peptide vaccine plus IL2, and M-vax plus IL2, improved patient OS. T-VEC got the approval by the FDA as a treatment for advanced melanoma. While most vaccine candidates have exhibited acceptable safety profiles and induced measurable immune activation, their clinical efficacy as monotherapies has typically been insufficient to warrant further large-scale investigation. Notably, enhanced clinical outcomes, including superior survival and objective response rates, have been consistently documented when vaccines are administered alongside other immunomodulatory agents. This collective evidence strongly suggests that the future of melanoma immunotherapy lies in combination strategies. It is anticipated that within the next decade, vaccines will be strategically deployed to prime and amplify tumor-specific T cells, concurrently with agents designed to counteract key mechanisms of tumor-mediated immune resistance.

## Supplementary material

Supplementary Table 1 Phase I/II trials to assess antitumor vaccines in patient with melanoma.

## Glossary

APC: Antigen presenting cell
BCG: Bacillus Calmette-Guérin
CR: Complete response
CTLA-4: Cytotoxic T-lymphocyte-associated protein 4
DCVs: Dendritic cell vaccines
DCs: Dendritic cells
DFS: Disease-free survival
DMFS: Distant metastasis-free survival
DRR: Durable response rate
GM-CSF: Granulocyte-macrophage colony-stimulating factor
GAd: Great Apes
HuIgG1 antibody DNA vaccine: HuIgG1 antibody DNA vaccine
HLA: Human leukocyte antigen
ICI: Immune checkpoint inhibitors
IFA: Incomplete Freund’s adjuvant
IDO: Indoleamine 2,3-dioxygenase
MHC: Major histocompatibility complex
MVA: Modified Vaccinia Ankara
NY-ESO-1: New York Esophageal squamous cell carcinoma-1
NR: Not reach
ORR: Objective response rate
OS: Overall survival
PR: Partial response
PBMCs: Peripheral blood mononuclear cells
PD-1: Programmed cell death protein 1
PFS: Progression-free survival
PD: Progressive disease
RFS: Relapse-free survival
SD: Stable disease
T-VEC: Talimogene laherparevec
TLR: Toll-like receptor
TAA: Tumor associated antigen
TLPO: Tumor lysate particle only
TILs: Tumor-infiltrated-lymphocytes
FDA: United States food and drug administration
