# Supplementary material for: Vaccines as treatment for melanoma: an update review
Source: Front Immunol. 2026 Jun 9;17:1724490. doi: 10.3389/fimmu.2026.1724490 (PMC13287066; doi:10.3389/fimmu.2026.1724490)
Supplement: Supplementary Table 1 — Phase I/II trials to assess antitumor vaccines in patient with melanoma. [file Table1.docx]

**Supplementary Table S1, phase I/II trials to assess antitumor vaccines in patient with melanoma**

| **Vaccine type** | **Study title** | **Phase** | **Patients enrolled** | **Results and/or comment** |
| --- | --- | --- | --- | --- |
| Tumor cell vaccines and dendritic cell vaccines | Phase II trial of vaccination with autologous, irradiated melanoma cells engineered by adenoviral mediated gene transfer to secrete granulocyte-macrophage colony stimulating factor in patients with stage III and IV melanoma (NCT00809588) | II | 61 | Vaccination with autologous melanoma cells secreting GM-CSF augments antitumor immunity in stage III and IV patients with melanoma, is safe, and demonstrates disease control. |
|  | An Open-Label Study of the Safety and Efficacy of Tag-7 Gene-Modified Tumor Cells-Based Vaccine in Patients with Locally Advanced or Metastatic Malignant Melanoma or Renal Cell Cancer. (NCT04180774) | I/II | 68 | GMV showed high results in carefully selected patients with low ISF (TGF-β1, IL-10, and VEGF) production. The method should be further investigated in patients with FP. |
|  | Cytokine network analysis of immune responses before and after autologous dendritic cell and tumor cell vaccine immunotherapies in a randomized trial (NCT004936930) | II | 42 | DCV induced a more effective immune response than that induced by TCV, and these immune responses were associated with improved survival. DCV was associated with innate, T1/T17, and T2 responses while TCV was only associated with innate and T2 responses. |
|  | An Update of Cutaneous Melanoma Patients Treated in Adjuvancy With the Allogeneic Melanoma Vaccine VACCIMEL and Presentation of a Selected Case Report With In-Transit Metastases (CASVAC-0401) | II | 30 | VACCIMEL plus BCG and GM-CSF is an effective treatment in adjuvancy for stages IIB, IIC, and III cutaneous melanoma patients, and it is compatible with subsequent treatments with ICKi. |
|  | Phase II Study of Adjuvant Immunotherapy with the CSF-470 Vaccine Plus Bacillus Calmette-Guerin Plus Recombinant Human Granulocyte Macrophage-Colony Stimulating Factor vs Medium-Dose Interferon Alpha 2B in Stages IIB, IIC, and III Cutaneous Melanoma Patients: A Single Institution, Randomized Study. (NCT01729663) | II/III | 31 | CSF-470 vaccine plus BCG plus GM-CSF can significantly prolong, with lower toxicity, the DMFS of high-risk CM pts with respect to medium-dose IFN-α2b. |
|  | Prospective, randomized, double-blind phase 2B trial of the TLPO and TLPLDC vaccines to prevent recurrence of resected stage III/IV melanoma: a prespecified 36-month analysis. (NCT02301611) | IIb | 187 | The TLPO and TLPLDC (without G- CSF) vaccines were associated with improved DFS and OS in this clinical trial. Given production and manufacturing advantages, the efficacy of the TLPO vaccine will be  confirmed in a phase 3 trial. |
|  | Randomized phase II trial of lymphodepletion plus adoptive cell transfer of tumor-infiltrating lymphocytes, with or without dendritic cell vaccination, in patients with metastatic melanoma. (NCT00338377.) | II | 18 | Although more patients showed a clinical response to TIL+DC therapy, this study was not powered to resolve differences between groups. |
| Peptide vaccines and protein vaccines | Clinical Activity of Combined Telomerase Vaccination and Pembrolizumab in Advanced Melanoma: Results from a Phase I Trial. (NCT03538314) | I | 30 | Encouraging safety and preliminary efficacy were observed. |
|  | Characterization of the T cell receptor repertoire and melanoma tumor microenvironment upon combined treatment with ipilimumab and hTERT vaccination. (NCT02275416) | I/IIa | 12 | Clinical responses were observed irrespective of established predictive biomarkers for checkpoint inhibitor efficacy, indicating an added benefit of the vaccine-induced T cells. |
|  | A phase 1 study of NY-ESO-1 vaccine + anti-CTLA4 antibody Ipilimumab (IPI) in patients with unresectable or metastatic melanoma. (NCT01810016) | I | 8 | Detection of T cell responses to NY-ESO-1 ex vivo in most patients suggests that IPI may have enhanced those responses. Proliferating intratumoral CD8+ T cells increased after vaccination plus IPI suggesting favorable impact of IPI plus NY-ESO-1 vaccines on the TME. |
|  | Combined Vaccination with NY-ESO-1 Protein, Poly-ICLC, and Montanide Improves Humoral and Cellular Immune Responses in Patients with High-Risk Melanoma. (NCT01079741) | I,II | 43(I:13, II:30) | Vaccination with NY-ESO-1 administered with poly-ICLC and montanide is superior in inducing integrated antibody and CD4þ T-cell responses in the majority of the patients, and CD8þ T-cell responses in some patients, compared with poly-ICLC alone.vaccination with NY-ESO-1 protein and poly-ICLC with or without montanide safely induces integrated NY-ESO-1– specific humoral and CD4þ T-cell responses. Both regimens were well tolerated, and at last follow-up, most phase II patients were alive without clinical evidence of disease. |
|  | Phase I/II trial of a long peptide vaccine (LPV7) plus toll-like receptor (TLR) agonists with or without incomplete Freund's adjuvant (IFA) for resected high-risk melanoma. (NCT02126579) | I/II | 50 | The LPV7 vaccine is safe with each of seven adjuvant strategies and induced T cell responses to CD8 MEPs ex vivo in a subset of patients but did not enhance IRRs compared with prior vaccines using short peptides. Immunogenicity was supported more by IFA than by TLR agonists alone and may be enhanced by polyICLC plus IFA. |
|  | A phase 1/2 trial of an immune-modulatory vaccine against IDO/PD-L1 in combination with nivolumab in metastatic melanoma. (NCT03047928) | I/II | 30 | These clinical efficacy and favorable safety data support further validation in a larger randomized trial to confirm the clinical potential of this immunomodulating approach. |
|  | Multipeptide vaccines for melanoma in the adjuvant setting: long-term survival outcomes and post-hoc analysis of a randomized phase II trial. (NCT00118274) | II | 167 | Immunogenicity of both vaccines but that CD8 T cell responses to12MPwere lowerwhentetwas replaced with6MHP. benefit was experienced only bymale patients. support the value of adding cognate T cell help to cancer vaccines and also suggest a need to assess the impact of patient sex on immune therapy outcomes. |
|  | Phase I/II clinical trial of a helper peptide vaccine plus PD-1 blockade in PD-1 antibody-naïve and PD-1 antibody-experienced patients with melanoma (MEL64) (NCT02515227) | I/II | 22 | Treatment with the 6MHP vaccine plus pembrolizumab was safe, increased intratumoral lymphocytes, and induced T cell Rsps associated with prolonged OS. The low T cell Rsp rate in PD- 1 Ab- experienced participants corroborates prior murine studies that caution against delaying cancer vaccines until after PD- 1 blockade. The promising objective response rate and OS in PD- 1 Ab- naïve participants support consideration of a larger study in that setting. |
|  | Immunogenicity in humans of a transdermal multipeptide melanoma vaccine administered with or without a TLR7 agonist. (IRB- HSR 11490) | I | 28 | These data provide proof of principle for immunogenicity in humans of transdermal immunization using peptides in DMSO. |
|  | Clinical and Immunological Outcomes in High-Risk Resected Melanoma Patients Receiving Peptide-Based Vaccination and Interferon Alpha, With or Without Dacarbazine Preconditioning: A Phase II Study. (EudraCT no. 2008-008211-26) | II | 34 | The combination of peptide vaccination with IFN-α2b showed a minimal toxicity profile and resulted in encouraging RFS and OS rates. |
|  | A multipeptide vaccine plus toll-like receptor agonists LPS or polyICLC in combination with incomplete Freund's adjuvant in melanoma patients. (NCT01585350) | I | 51 | LPS and polyICLC are safe and effective vaccine adjuvants when combined with IFA. Contrary to the central hypothesis, IFA enhanced T cell responses to peptide vaccines when added to TLR agonists |
| Neoantigen vaccines | Personalized therapy with peptide-based neoantigen vaccine (EVX-01) including a novel adjuvant, CAF®09b, in patients with metastatic melanoma. (NTC03715985) | I | 5 | Personalized immunotherapy with neoantigens is a promising approach in cancer treatment, and precise identification of immunogenic neoantigens are required for effective neoantigen-based cancer immunotherapy. |
|  | Personal neoantigen vaccines induce persistent memory T cell responses and epitope spreading in patients with melanoma. (NCT01970358) | I | 8 | Long-term persistence of neoantigen-specific T cell responses following vaccination, with ex vivo detection of neoantigen-specific T cells exhibiting a memory phenotype.we detected evidence of tumor infiltration by neoantigen-specific T cell clones after vaccination and epitope spreading, suggesting on-target vaccine-induced tumor cell killing. |
|  | Combined TCR Repertoire Profiles and Blood Cell Phenotypes Predict Melanoma Patient Response to Personalized Neoantigen Therapy plus Anti-PD-1. (NCT02897765) | Ib | 26 | Vaccine-induced T cells persist over time, exhibit cytotoxic potential, and can migrate to tumors. Epitope spread and major pathologic tumor responses were detected following vaccination. |
| Viral, bacterial and fungal vaccines | Phase I Trial of Viral Vector-Based Personalized Vaccination Elicits Robust Neoantigen-Specific Antitumor T-Cell Responses. (NCT04990479) | Ib | 6 | Amplify and broaden the repertoire of tumor-reactive T cells to empower a diverse, potent, and durable antitumorimmune response. |
|  | An intra-patient placebo-controlled phase I trial to evaluate the safety and tolerability of intradermal IMM-101 in melanoma. (NCT01308762) | i | 18 | IMM-101 is safe and well tolerated and there is a rationale for studying IMM-101 at a nominal 1.0-mg dose to complement conventional cytotoxic therapy for patients with advanced cancer |
| Nucleic acid vaccines | An RNA vaccine drives immunity in checkpoint-inhibitor-treated melanoma. (NCT02410733) | I | 56 | The general utility of non-mutant shared tumour antigens as targets for cancer vaccination. |
|  | Individualised neoantigen therapy mRNA-4157 (V940) plus pembrolizumab versus pembrolizumab monotherapy in resected melanoma (KEYNOTE-942): a randomised, phase 2b study (NCT03897881) | IIb | 157 | Adjuvant mRNA-4157 plus pembrolizumab prolonged recurrence-free survival versus pembrolizumab monotherapy in patients with resected high-risk melanoma and showed a manageable safety profile. |
|  | Intradermal Naked DNA Vaccination by DNA Tattooing for Mounting Tumor-Specific Immunity in Stage IV Melanoma Patients: A Phase I Clinical Trial. (NL.20284.000.08) | Ia | 9 | Developed DNA vaccine, applied using a novel intradermal applicationstrategy, can be administered safely. |
|  | Targeting gp100 and TRP-2 with a DNA vaccine: Incorporating T cell epitopes with a human IgG1 antibody induces potent T cell responses that are associated with favourable clinical outcome in a phase I/II trial (NCT01138410) | I/II | 35 | SCIB1 is well tolerated and stimulates potent T cell responses in melanoma  patients. |
